# Constructing more informative plant–pollinator networks: visitation and pollen deposition networks in a heathland plant community

**DOI:** 10.1098/rspb.2015.1130

**Published:** 2015-09-07

**Authors:** G. Ballantyne, Katherine C. R. Baldock, P. G. Willmer

**Affiliations:** 1 School of Biological Sciences, University of St Andrews, Harold Mitchell Building, St Andrews KY16 9TH, UK; 2 School of Biological Sciences, University of Bristol, Life Sciences Building, Bristol BS8 1TQ, UK; 3 Cabot Institute, University of Bristol, Bristol BS8 1UJ, UK

**Keywords:** pollination, network, species interaction, pollinator effectiveness, pollinator importance, specialization

## Abstract

Interaction networks are widely used as tools to understand plant–pollinator communities, and to examine potential threats to plant diversity and food security if the ecosystem service provided by pollinating animals declines. However, most networks to date are based on recording visits to flowers, rather than recording clearly defined effective pollination events. Here we provide the first networks that explicitly incorporate measures of *pollinator effectiveness* (PE) from pollen deposition on stigmas per visit, and *pollinator importance* (PI) as the product of PE and visit frequency. These more informative networks, here produced for a low diversity heathland habitat, reveal that plant–pollinator interactions are more specialized than shown in most previous studies. At the studied site, the specialization index 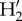 was lower for the visitation network than the PE network, which was in turn lower than 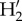 for the PI network. Our study shows that collecting PE data is feasible for community-level studies in low diversity communities and that including information about PE can change the structure of interaction networks. This could have important consequences for our understanding of threats to pollination systems.

## 1. Introduction

Given current concerns over pollinator declines and the resultant impact on both food production and plant diversity, we need to understand how pollinator deficits could affect pollination services for both crops and wild plants [1–3]. The field of plant–pollinator networks is flourishing, with increasing numbers of studies using interaction web or network approaches, and more sophisticated analytical methods being developed to examine interactions between plants and their potential pollinators [4–6]. Most networks quantify plant–pollinator interactions as numbers of animal visits to flowers (‘visitation’ or ‘flower-visitor’ networks), though a few ‘pollen-transport’ networks demonstrate which visitors are potentially important pollinators, based on quantity and species composition of pollen loads carried [7,8] (although this pollen may have many fates other than deposition on stigmas [9]).

While these studies examine community-level interactions among plants and visitors, most do not distinguish between mere flower visitors and effective pollinators. An animal visit to a flower does not necessarily constitute a pollination event, which requires a visitor that transfers pollen from anthers of one flower to stigmas of conspecifics. Networks are increasingly used as tools to assess effects of introduced and/or invasive species [10,11], potential extinction rates [12,13] or resilience to anthropogenic factors such as climate or landscape change [6,14,15], all of which have implications for conservation strategies [16]. Visitation networks provide essential information on resource use by flower visitors. However, when pollination itself is being investigated it is crucial to know which apparently legitimate visitors are depositing significant conspecific pollen on stigmas and thus potentially effecting pollination.

All pollination biologists are fully aware that ‘visitor’ is not a synonym for ‘pollinator’; some visitors are purely cheaters, removing pollen or nectar without pollinating flowers, while other (non-cheating) visitors may be detrimental (e.g. causing stigma blockage with heterospecific pollen) and/or have low effectiveness. Authors have varied in how far they allow for these problems. Some have assumed the most effective pollinators were the most frequent flower visitors [17,18] but this has been strongly criticized (e.g. [19]). Others aimed to improve accuracy by recording only visitors making contact with floral reproductive organs, thus hoping to exclude illegitimate visitors [20,21]; however, high-speed video recordings of visitors to *Clerodendrum trichotomum* [22] reveal significant differences in behaviour between visitor species not visible to the naked eye, showing that the most frequent visitors are rarely most efficient at contacting anthers or stigmas. Other studies have explicitly incorporated additional data, such as visit patterns (number, or rate, or duration, or visits per plant), pollen transport (amount carried on body, or distance moved), or resultant seed-set [13,23,24]. Recognizing the limitations of all these approaches, a few authors (e.g. [8,10,24,25] refer only to ‘visitation networks’ rather than implying that they are recording effective pollination. Nevertheless, others assert that most visitors are functionally equivalent in their pollen-moving ability (e.g. [26]), or that visitor frequency is an acceptable surrogate (e.g. because variation in frequency ‘overwhelms per visit effectiveness’ [27]).

Analyses of visitation data have concluded that ‘pollination networks’ are relatively robust, with nestedness and connectance invariant across years, whether working with simulations [12,13] or empirical datasets [28,29], and that a moderate level of extinctions could therefore be tolerated. They also conclude that most flower-visiting animals are generalized in their flower choices, with specialist interactions being rare. But if some common flower visitors in fact contribute little conspecific pollen transfer, these analyses could give misleading perspectives for approaches to plant conservation and thus ecosystems as a whole [30,31]. Recognizing this, a more realistic representation of community interactions is desirable [32–34].

Thus, there is a need to incorporate functionality measures indicating effective pollination into community studies and thence into networks. Here we compare traditional flower-visitor networks with novel pollinator effectiveness (PE) networks, following Ne'eman *et al.* [35] and King *et al.* [33] in defining PE as single visit deposition (SVD: number of conspecific pollen grains deposited on a virgin stigma during a single visit by a particular animal). We then create pollinator importance (PI) networks by combining PE with visit frequency for each flower visitor interaction.

Some early studies were exemplary in using pollen deposition onto stigmas to compare visitors with single species (e.g. [36,37]). We have since demonstrated [33] that SVD can be compared between visitors and flowers with very different morphologies, and that it varies significantly between visitor species. Other recent studies have compared PE for visitors with single flower species or a few congeneric species (e.g. [38,39]). Here we show that SVD is feasibly incorporated in community-level field studies with low plant diversity, and present the first explicit PE and PI networks, specifically addressing the following questions: (i) how does PE (SVD) vary between different flower visitors? We predict there will be significant variation in the effectiveness of pollen deposition by different insect species, due to variation in size and behaviour. (ii) Does visit type influence PE? Visitation by pollen-collecting insects, contacting anthers and usually stigmas, is predicted to result in higher pollen deposition compared with nectar-foragers. Legitimate visitation should also result in higher pollen deposition than robbing visits, as nectar robbers/thieves (often visiting via basal holes in the corolla) are less likely to contact stigmas. (iii) How do PE and PI networks compare with flower visitor networks? Here we predict that incorporating more detailed information concerning the nature of the visit will yield more specialized networks.

## 2. Material and methods

### (a) Study site and species

Fieldwork was carried out at Hyde Heath, Dorset (50°43·7′ N 2°07·2′ W) from June to August 2013, and in May 2014 to incorporate the early flowering *Ulex europaeus*. This site, covering around 600 ha, offers a low diversity community, for which visitation data have already been published [40]. It is, therefore, an ideal habitat to demonstrate the feasibility of the approach before addressing more complex (higher diversity) communities. The site's flora is almost exclusively heather (*Erica tetralix*, *E. cinerea* and *Calluna vulgaris*) and gorse (*U. europaeus* and *U. minor*). *Polygala serpyllifolia* is present at very low density, but received no visits during the study. For additional information on floral phenology, abundance and reward levels, see electronic supplementary material, S1.

### (b) Pollen deposition

To obtain SVD data, our measure of PE, flower buds were bagged each evening, weather permitting, throughout the study period; this involved covering whole *E. tetralix*, *E. cinerea* and *C. vulgaris* plants or groups of *Ulex* buds with mesh. The mesh was carefully removed the following morning, once flowers had opened and virgin stigmas identified on flowers that had opened overnight using a hand lens.

Individual flowers were then observed until they received their first visit. We obtained SVD data from a minimum of 90 individual flowers per plant species and up to 350 flowers for the more common species (table 1). Visitor identity, time and duration of visits and visitor behaviour on the flower were recorded, including resources collected and nature of collection: legitimate (via the corolla mouth) or robbing (chewing a hole through the corolla). Where identity to genus was not obvious the visitor was photographed and/or caught for later identification. *Bombus* could be identified to species, although the common *B. terrestris* and *B. lucorum* are difficult to distinguish in the field [41] so were grouped as *B. terrestris/lucorum* (cf. [40]). Owing to small sample sizes, other visitors were grouped according to taxonomy and/or size. Halictid bees (almost all *Lasioglossum*) were pooled, while *Andrena* and *Colletes* (similarly sized bees) were pooled as ‘other solitary bees’. For hoverflies, *Episyrphus balteatus* and *Eupeodes corollae* visits were identified specifically, with other less common genera grouped as ‘large hoverflies’ (*Eristalis*, *Syrphus*, *Helophilus* and *Volucella*) and ‘small hoverflies’ (*Stratiomys*, *Platycheirus* and *Meliscaeva*). Surveys continued on each day until there were no more bagged flowers left to sample or visitation rate had decreased to a very low level (no visits recorded for 1 h). Most data were collected between 08.00 h and 15.00 h in dry conditions with low winds. On warmer July days sampling occurred until 19.00 h, matching visitor activity patterns.

**Table 1.** Mean SVD values (numbers of conspecific pollen grains deposited on stigmas) for different visitors to each plant species; means ± s.e., with *n* (number of visits recorded) in parentheses. Mean controls rounded to the closest whole number and subtracted from all SVD values shown for that plant species. ‘Flower hours’ is calculated as the product of length of time flowers were watched and the number of flowers watched in each hour.

	*Erica tetralix*	*Erica cinerea*	*Calluna vulgaris*	*Ulex minor*	*Ulex europaeus*
observation time (flower hours)	2070	2268	1326	618	168
control stigmas	0.31 ± 0.18 (13)	0.47 ± 0.22 (12)	8.13 ± 2.02 (23)	8.36 ± 1.29 (14)	8.00 ± 2.68 (9)
visitor groups					
bees					
*Bombus terrestris/lucorum*	11.59 ± 1.54 (271)	35.33 ± 3.39 (228)	21.02 ± 2.10 (135)	45.36 ± 8.28 (33)	43.10 ± 4.35 (40)
*Bombus pascuorum*	29.29 ± 12.74 (14)	—	—	43.13 ± 7.44 (31)	45.55 ± 7.33 (11)
*Bombus lapidarius*	28.50 ± 10.50 (2)	32.70 ± 7.48 (33)	22.41 ± 4.60 (17)	63.00 ± 8.81 (48)	48.11 ± 11.80 (9)
*Bombus hortorum*	—	—	—	—	24.20 ± 8.29 (5)
*Bombus jonellus*	55.27 ± 16.27 (11)	47.83 ± 23.26 (6)	—	—	—
*Apis mellifera*	2.94 ± 0.51 (77)	21.79 ± 4.93 (56)	25.07 ± 2.06 (175)	—	17.67 ± 3.65 (12)
Halictidae	25.80 ± 11.84 (5)	19.17 ± 3.62 (23)	—	—	—
other solitary bees	—	—	6.33 ± 4.11 (6)	82.33 ± 20.84 (9)	67.00 ± 19.54 (6)
flies					
*Episyrphus*	—	—	19.17 ± 8.50 (12)	21.00 ± 20.00 (3)	—
*Eupeodes*	—	3.00 ± 2.08 (3)	7.75 ± 7.42 (4)	—	—
large hoverflies	8.00 ± 4.90 (4)	—	6.00 ± 4.02 (4)	3 (1)	—
small hoverflies	—	11 ± 4 (2)	7.83 ± 7.83 (6)	3 (1)	—
Muscidae	—	—	22.37 ± 6.17 (19)	—	—
soldier fly	**—**	**—**	1 (1)	**—**	**—**
other					
ants (*Lasius*)	5.50 ± 4.97 (14)	—	—	—	—
Lepidoptera	—	—	—	—	9 (1)
total visits recorded	398	351	379	126	84

After each insect visit, the stigma from that flower was removed with clean tweezers and dabbed onto a cube of fuchsin agar gel, thus removing and staining the pollen. Use of a hand lens ensured all pollen grains had been removed. Gels were melted on microscope slides under coverslips, and all conspecific and heterospecific pollen grains deposited were counted by light microscopy (×100 or ×400). Pollen morphology of *Erica* species varies little, so absolute distinction of *E. tetralix* and *E. cinerea* pollen was not always possible, but errors would be reduced by their differing flowering phenology with only *E. cinerea* still abundant into August (see electronic supplementary material, S1).

To account for pollen found on stigmas due to opening of the flower and/or handling and bagging procedures, control stigmas were also sampled for each plant species (8–12 per species). Stigmas were removed from newly uncovered virgin flowers before a visit took place and checked for pollen as above. Mean control values for each species were subtracted from SVD values obtained from individual visits.

### (c) Floral visitation

The visits to flowers during SVD observations provided data used to construct visitation networks. Total observation time per plant species in the visitation dataset varied because of the diversity of flower visitors, length of flowering season and floral abundance within the habitat; hence the very common heather flowers, with a wide range of visitors, were observed for longer than the less common gorse flowers.

### (d) Network construction

The data were used to construct the following networks: (i) visitation (V) network, using the frequency of interaction between visitor groups and plant species. As network metrics may be strongly influenced by methodology of data collection [42], this network was constructed using data collected during stationary PE observations. As sample sizes varied among species, visit frequency for a plant–visitor group interaction was calculated as a proportion of the total number of visits by all visitor groups, so that interaction bar widths sum to 1 for each plant species, removing bias from variation in sampling effort. (ii) PE network, using mean SVD values for each visitor group to each plant species. Pollen production and hence deposition varied greatly between plant species; to remove bias from this variation, the PE interaction between each visitor group and plant species was calculated as a proportion of the total SVD for that plant species (i.e. total pollen grains deposited across all SVD observations). (iii) PI network, combining data from V and PE networks. PI for each interaction was calculated as the product of total visitation frequency (using stationary observation data) and mean PE for that visitor group. Again biases were accounted for by using PI values for each visitor group/plant species interaction calculated as a proportion of the total PI summed across all visitor groups for that plant, so that all interaction bar widths for each plant species sum to 1.

### (e) Data analysis

#### (i) The effect of visitor identity on pollen deposition

Parametric statistics could not be used to analyse pollen deposition data as residuals were not normally distributed given the high proportion of zeros in the dataset. Therefore, non-parametric Kruskal–Wallis tests were used to compare SVD among visitor groups for each plant species. *Post hoc* tests (pairwise Wilcoxon rank sum tests) tested for significant differences among visitor groups. A Holm–Bonferroni correction for multiple testing was used for both steps. (Results from a more complex analysis using a negative binomial GLM, supporting the results obtained from these tests, can be found in electronic supplementary material, S2.)

#### (ii) The effect of individual visitor behaviour on pollen deposition

Kruskal–Wallis tests determined whether different visitor behaviours (pollen versus nectar-collecting, and legitimate versus robbing visits) yielded different SVD levels. Visitors to *Ulex* species only gathered pollen, and were, therefore, excluded from this comparison, while robbing behaviour was only observed for *Erica* species.

#### (iii) Comparing visitation networks with pollinator effectiveness and pollinator importance networks

Interaction networks were analysed using the bipartite package (v. 2.05 [43], in R v. 3.0.1 [44]) and a number of network metrics extracted. The relevance and/or utility of common network metrics have been much debated (e.g. [45,46]). Following an emerging consensus, our analyses focused on a key range of metrics. We used 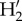 to measure network specialization as it best represents the level of interaction selectiveness by estimating the deviation of observed interaction frequencies from expected values from a null distribution of interactions. 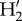 is based on weighted links and, therefore, robust against sampling effort [47]. 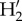 ranges from 0 (extreme generalization) to 1 (perfect specialization). We used *d*′ to measure of species-level specialization, which measures the exclusivity of interactions that individual species take part in [48]. This is the most biologically informative measure of visitor specialization in resource choice in a visitation network, and most relevant predictor of specialization in pollination for a plant in a PI network. As the matrix data are proportional, all values were multiplied by 1000 before calculating this metric. Species strength, on the other hand, measures the sum of an individual species dependencies (relative interaction weights) within a network [49]; it is most biologically informative for plants in a visitation network, as resource use of these species by visitors is measured, and for visitors in a PI network, where potential pollination quality is measured. We compared *d*' and species strength between V and PI networks using paired *t*-tests (data conformed to parametric test assumptions and, as *d*′ values are proportions, these were arcsine square-root transformed before testing). We did not statistically compare *d*′ and species strength of the PE network as these did not include a measure of visitor interaction frequency. Generality of visitor species, together with generality of plant species (the latter is also termed vulnerability because of its use in the food web literature, describing the vulnerability of prey to predation [50]), measures the mean numbers of species a plant or visitor group directly interacts with, weighted to account for sample size. Interaction evenness measures homogeneity in interaction frequencies, which reaches 1 when the number of interactions between plants and visitor groups is uniformly distributed, and is inversely related to network stability [51]. Nestedness, weighted by sample size (WNODF [52]), estimates linkage structure. Here 1 indicates perfect nestedness and 0 perfect chaos, with greater nestedness conferring higher stability in mutualistic networks [53]. Recent studies (e.g. [54]) indicate that nestedness may only be a secondary indicator of stability; however, the primary driver, degree distribution (linkage density), cannot be accurately calculated for small networks [55].

## 3. Results

### (a) The effect of visitor identity on pollen deposition

A total of 1338 insect–flower interactions were observed, all providing SVD data (table 1). Sixty-seven per cent of observations were *Bombus* spp., 24% *Apis,* 4% solitary bees, 3% hoverflies and 1% other rare visitors (butterflies, ants, other Diptera). Bumblebees were the main flower visitors (over 75% of visits) for all plant species except *C. vulgaris*, where *Apis* was most common (45% of visits). Of all *Bombus* visits, 79% were made by the *B. terrestris/lucorum* group.

All visitor groups showed high variation in SVD (table 1). There were no significant differences in SVD among visitor groups for *E. cinerea, C. vulgaris, U. minor* or *U. europaeus* following correction for multiple testing. Only visitors to *E. tetralix* differed significantly in SVD (*χ*^2^ = 45.2, d.f. = 7, *p* < 0.001); here *post hoc* tests revealed that *Bombus jonellus* deposited significantly more pollen onto stigmas than *B. terrestris/lucorum* (*W* = 2476, *p* < 0.001), *Apis* (*W* = 75.5, *p* < 0.001) and *Lasius* (*W* = 16.5, *p* < 0.001), while *B. terrestris/lucorum* deposited significantly more pollen onto stigmas than *Apis* (*W* = 7153, *p* < 0.001). When visitor groups with fewer than five visits were excluded from analyses the results were found to be the same. Comparable results were obtained using negative binomial GLMs (electronic supplementary material, S2).

### (b) The effect of visitor behaviour on pollen deposition

**Figure 1.**
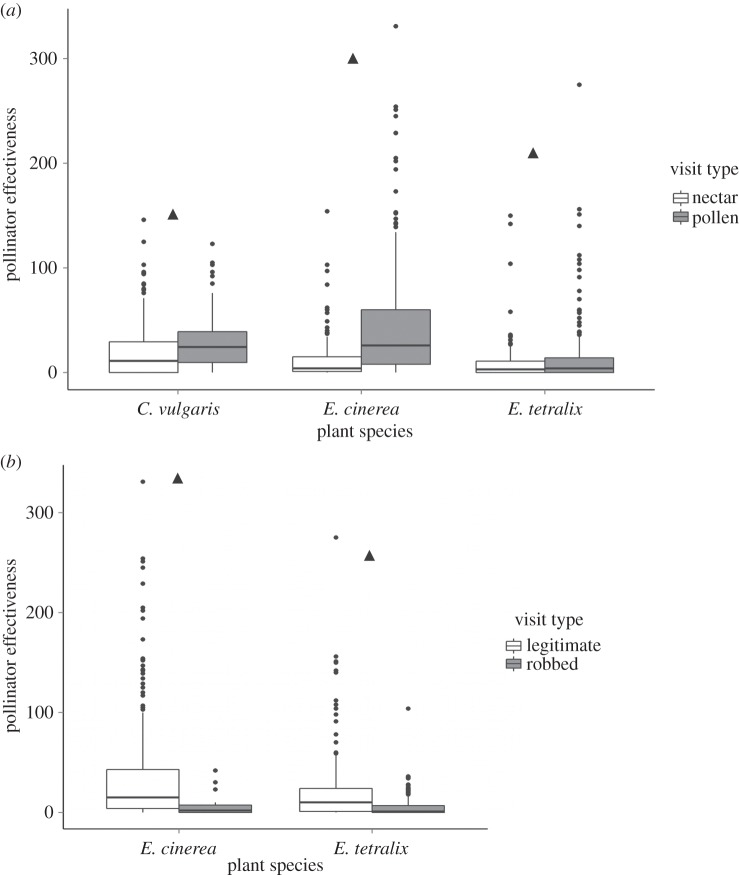
Single visit deposition (PE) of conspecific pollen: (*a*) pollen and nectar-foragers; (*b*) legitimate and robbing flower visitors. Median, interquartile range and outliers for individual visits are shown for each plant species. Significant differences marked with triangle.

Pollen-foraging visitors had significantly higher PE than nectar-foraging visitors for *E. tetralix* (figure 1*a*; *χ*^2^ = 39.81, d.f. = 1, *p* < 0.001), *E. cinerea* (*χ*^2^ = 68.16, d.f. = 1, *p* < 0.001) and *C. vulgaris* (*χ*^2^ = 23.62, d.f. = 1, *p* < 0.001). A pollen-forager was more likely to receive pollen on its body and thus to move conspecific pollen between flowers. This result is unlikely to be skewed by visitor size (cf. [56]), as large bumblebees often collected only nectar (occasional male visitors doing so exclusively), and smaller visitors (small hoverflies, halictid bees) nearly always directed their mouthparts to the anthers (which are extended away from the nectaries in all three species) and collected just pollen.

As expected, legitimate visitors were usually visibly coated in pollen and deposited significantly more pollen than visitors that robbed flowers of both *E. tetralix* (figure 1*b*; *χ*^2^ = 53.7, d.f. = 1, *p* < 0.001) and *E. cinerea* (*χ*^2^ = 12.54, d.f. = 1, *p* < 0.001). Legitimate visitors are more likely to contact anthers and stigmas than basal robbers, and will, therefore, more readily pick up pollen and deposit it on stigmas.

### (c) Comparing visitation, pollinator effectiveness and pollinator importance networks

**Figure 2.**
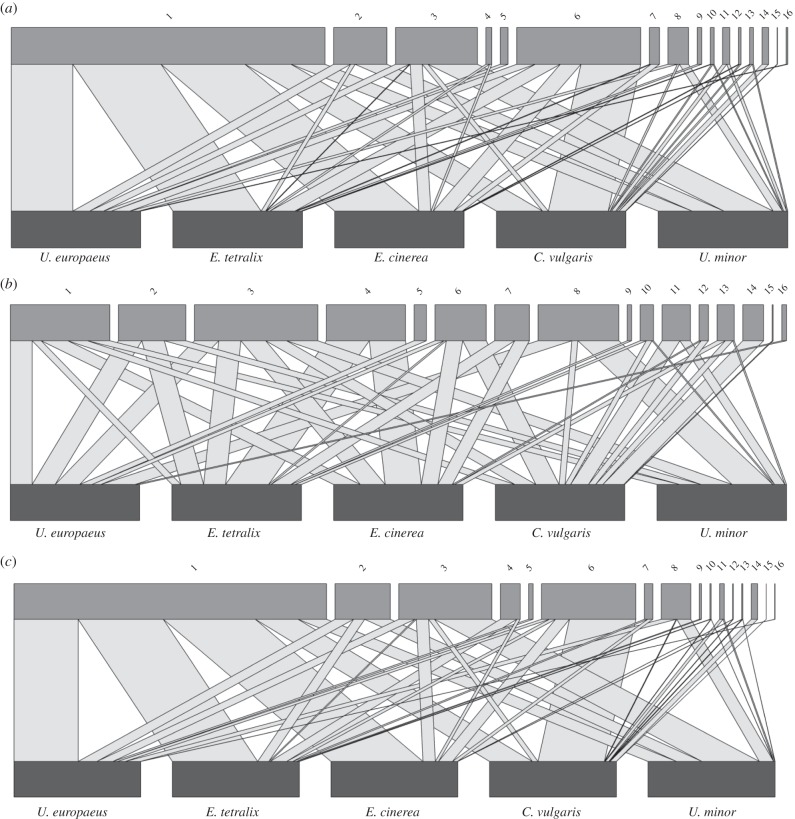
Bipartite networks illustrating (*a*) flower visitation, from stationary observations; (*b*) pollinator effectiveness of visitors (mean SVD); (*c*) pollinator importance of visitors, combining the data from (*a*) and (*b*). Key code, visitor group: 1, *Bombus terrestris/lucorum*; 2, *Bombus pascuorum*; 3, *Bombus lapidarius*; 4, *Bombus jonellus*; 5, *Bombus hortorum*; 6, *Apis mellifera*; 7, Halictidae; 8, other solitary bees; 9, large hoverflies; 10, *Episyrphus*; 11, *Eupeodes*; 12, small hoverflies; 13, Muscidae; 14, ants (*Lasius*); 15, Lepidoptera; 16, soldier fly.

Bipartite networks constructed from the three different datasets are shown in figure 2*a–c*, and corresponding network metrics in table 2. Figures 2*b*,*c* allow direct comparisons of individual visitor performance. For example, some visitors such as *B. jonellus* visiting *Erica* spp., or solitary bees visiting *U. minor*, do deposit high numbers of pollen grains but have low PI levels (figure 2*c*) because of their low visitation rates. By contrast, others such as *B. terrestris/lucorum* deposit average numbers of pollen grains, but their high visitation rates result in much higher PI values.

**Table 2.** Network metrics for visitation, pollinator effectiveness (PE) and pollinator importance (PI) networks.

metric	network type		
	visitation	pollinator effectiveness (SVD)	pollinator importance
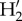	0.305	0.341	0.365
interaction evenness	0.663	0.780	0.643
weighted nestedness	0.108	0.041	0.179
generality			
visitors	3.764	3.195	3.561
plants	3.724	6.174	3.392

The V network was reasonably generalized (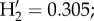 figure 2*a*), whereas the PE network based on SVD data was slightly more specialized (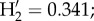 figure 2*b*). When stationary visitation and SVD data were combined, the resulting PI network was more specialized than both SV and PE networks (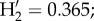 figure 2*c*). These results suggest that the addition of pollen deposition data (PE) to visitation data to give PI values can produce an increase in network specialization.

The other metrics show less clear trends among the three types of network. Interaction evenness varied little, though slightly higher for the PE network (table 2). It is likely that the PE network would have higher stability, as there is less variation in pollen deposition than in visitation rate. Weighted nestedness was lowest for the V network and highest for the PI network. This follows the same trend as for 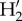 and suggests that the PI network had potentially higher stability (although given concerns regarding nestedness values for relatively small network sizes, this issue should be treated with caution). Visitor generality did not greatly differ among visitor groups, which is unsurprising given the limited pool of plant species that could be visited. Plant generality was higher in the PE network than in the V and PI networks. Most visitors to a particular plant species deposited similar amounts of pollen per visit (table 1), whether they were frequent or infrequent visitors, so that all contributed to this weighted metric. Both plant and visitor generality were lower in the PI network than in the V network, as the importance of visitors depositing little pollen was reduced.

There were no significant differences in species-level specialization *d*′ between V and PI networks for plants (*t* = 1.677, d.f. = 4, *p* = 0.169) or visitors (*t* = 0.433, d.f. = 15, *p* = 0.671) (electronic supplementary material, S3). All visitors in the V network showed fairly low specialization. Plant species also had relatively low specialization levels, with *C. vulgaris* and *U. minor* showing the highest *d*' values of 0.370 and 0.372, respectively, in the PI network. There were also no significant differences in species strength between V and PI networks for plants (*t* = 0.001, d.f. = 4, *p* = 0.999) or visitors (*t* = 0.003, d.f. = 15, *p* = 0.997) (electronic supplementary material, S3). *C. vulgaris* was the most important contributor to link weighting with the visitor community, with the highest species strength in the visitation network (4.830), while *B. terrestris/lucorum* contributed the most to PI link weightings, with the highest species strength in the PI network (2.465).

Overall, incorporating information on PE into visitation networks results in relatively more specialized interactions and provides slightly more accurate measures of plant species exclusivity, and of the contribution of visitor species to pollination. These results proved to be robust against alterations in visitor groupings (electronic supplementary material, S4).

## 4. Discussion

Measuring the difference between visitation and pollination is a challenge for pollination ecologists. For the first time, we report values for pollen deposition onto stigmas for virtually all components of a plant–pollinator community and demonstrate that such data enhance the quality of flower visitor interaction studies by producing networks giving a more accurate estimate of PI.

### (a) Patterns of insect–flower interactions

The flowering plants in this low diversity heathland habitat were visited by similar insect species to those recorded at the same site by Forup *et al.* [40], dominated by *Bombus* spp. and *Apis*. Although bees deposited the greatest mean quantities of pollen grains on stigmas, deposition rates were highly variable, resulting in no differences in effectiveness between most visitor groups. For the three heather species this was not surprising as they have small, easily accessed flowers with only moderately specialized morphology. Visitation to the labiate keel flowers of both *Ulex* species is clearly limited by floral morphology and trait complementarity [57], translating into ‘forbidden links’ in the context of networks [58]. Only insects able to push apart the flag and keel petals will release the anthers, so flowers are almost exclusively visited by large-bodied bees (table 1). As inappropriate visitors mostly avoid *Ulex* flowers there is often little variation in pollen deposition by those that do visit. Some visitor species to plants with more specialized floral morphology may deposit no pollen onto stigmas, emphasizing the visitor/pollinator distinction.

While bees appeared to be effective pollinators for all five plant species, hoverflies always deposited relatively few pollen grains on stigmas. Their relatively short tongues compared with bees, combined with their habit of making little bodily contact with anthers or stigma, mitigate against pollen deposition in either heather or gorse, and their low visitation rates further decreased their importance as pollinators. They may be highly effective pollinators of more generalist blooms such as rape flowers [59] or of specific ‘hoverfly flowers’ (cf. [2]).

As predicted, visitors that robbed flowers were also poor pollinators as they seldom contacted the anthers or stigmas of flowers. We also found that pollen-collecting visitors deposited more pollen on stigmas than nectar-collecting visitors for plant species on which both pollen- and nectar-foragers were recorded (*C. vulgaris*, *E. cinerea* and *E. tetralix*). Pollen-collectors forage more actively among anthers, thus enabling more pollen to accumulate on their bodies. Our findings, therefore, match with other studies (e.g. [39]), although the precise effect of foraging behaviour on pollen deposition may vary with floral morphology (e.g. [60]).

Several visitor groups, including small hoverflies and butterflies, visited flowers infrequently. Considering the visitation data in isolation these insects could be interpreted as providing a ‘back-up’ pollination service, as proposed [61] and detected [40] in other studies. While a ‘back-up’ option may often be useful for plants (especially, if there is temporal variation in visitor numbers), taking SVD into account in this study revealed that these infrequent visitors deposited rather small amounts of pollen. This again demonstrates the importance of considering pollen deposition data alongside visitation studies.

### (b) Comparing visitation and pollen deposition networks

The type of data used in a flower visitor network affects network structure and will thus influence interpretation of the relationships among plants and pollinators in a given community. As we predicted, visitation networks potentially underestimate the levels of specialization among plants and their pollinators, although this needs confirmation for diverse communities in a range of habitats. Crucially, our data suggest that combining visitation data with SVD data as a measure of functionality to create PI networks can subtly change the network structure, increasing specialization, decreasing the corresponding generality of plant species and visitor groups, and providing potentially more accurate measures of exclusivity for plant species and of species strength for visitors. Previous studies that focused on visitor behaviour (and thus probable effectiveness as pollinators) also demonstrated that removing ineffective pollinators affected network properties (e.g. [25]).

Our study considers a community with low plant diversity, deliberately selected to test the hypothesis that including data on PE would affect network structure, so showing the feasibility of our approach. Construction of comprehensive PE and PI networks for large, complex plant communities would be more challenging. However, effects of including SVD data could be even more pronounced in such communities, with a greater range of floral morphologies (and thus potentially higher variability in pollen deposition), not least because PE and PI networks will more accurately represent the likely role of each visitor as a pollinator. For example, our SVD data support the visitation data in showing the importance of *Apis* as a pollinator of *C. vulgaris*, but *Apis* as a pollinator of *E. tetralix* from visitation alone would be greatly overestimated as it deposited little conspecific pollen on stigmas.

We recognize that incorporating SVD into interaction networks presents challenges. Firstly, it may not be possible to accurately identify pollen to species; for example, our *Erica* possessed similar pollen morphologies, and in habitats with higher numbers of congeneric plants this problem could be magnified. Secondly, not all plant species are self-compatible, and there is no easy way to differentiate self- from non-self-pollen. Emasculation of experimental flowers would prevent any deposition of self-pollen, but risks altering visitor behaviour and time spent on flowers, especially for pollen-foragers. Future SVD studies incorporating data on pollen tube growth and self-compatibility will help to differentiate self- and cross-pollen. Thirdly, while PI networks have the potential to describe pollination interactions more accurately than visitation networks alone, collecting data is more time-consuming. Resultant smaller sample sizes could then limit opportunities to split data reliably, for example, by time-slicing across the day or flowering season, and could thus restrict the usefulness of certain network metrics. Fourthly, the sampling method could introduce subtle temporal biases, as SVD data can only be collected for the first visitor to a virgin stigma; though sequential unbagging through a day can limit this effect. Finally, there is also potential bias due to the reward status of flowers at the time of unbagging. When experimental flowers are exposed early in the day they will have rewards similar to other newly opened flowers, whereas those uncovered later may have retained higher reward levels, potentially leading to greater attractiveness and longer visits relative to non-experimental flowers (e.g. [62]). This could result in diurnal variation in recorded SVD.

Despite these potential issues, we propose that information gained from incorporating SVD data to create PE and PI networks provides greater insight into the quality of interactions between plants and their potential pollinators, and brings the field of plant–pollinator networks much closer to the construction of true pollination networks. SVD measures both an animal's ability to pick up pollen in previous visits to the same plant species (thus incorporating the key aspect of visit constancy), and to accurately deposit it in the only place where it can germinate and potentially lead to fertilization. By using single visits, SVD largely avoids problems of stigmatic overload or saturation, and resultant pollen competition. Furthermore, it avoids the complications that measurements of seed- or fruit-set bring, where post-pollination factors may have major effects on reproductive outcomes that are essentially unrelated to pollination; though at a smaller scale, under more controlled conditions, the methods could be accompanied by studies of post-pollination processes, investigating resulting seed-set. Ongoing construction of PE and PI networks for more complex, species-rich communities will demonstrate the feasibility of our approach for a wider range of visitors and of floral morphologies.

### (c) Conclusion

The choice of methods used to sample potential pollinators in flower-visitation studies will depend on the questions being asked. Where the main interest is visitation and resource collection from the perspective of flower visitors, SVD data add little extra information. If the focus is on pollination and pollen transport among conspecific flowers, SVD as a measure of PE gives valuable insights from the perspective of the plant. The resulting more realistic PE and especially PI networks bring us a step closer to understanding pollination at the community level.

While we have shown somewhat greater levels of specialization in our PE and PI networks, at least from the plant's perspective, we do not imply that extreme specialization is common in plants, and all visitors to flowers should be considered as potential pollinators. Understanding how true pollination networks are structured is crucial to understanding community interactions and thus how to restore and conserve pollination services in the face of pollinator decline.

## Supplementary Material

Floral abundance surveys

## Supplementary Material

Further analysis of pollen deposition by visitors

## Supplementary Material

Species level specialisation and species strength for plant species and visitor groups

## Supplementary Material

Network metrics from alternative visitor groupings

## Data accessibility

Project data are available to view on Dryad http://dx.doi.org/10.5061/dryad.17pp3.
