# Supplementary material for: Constructing more informative plant–pollinator networks: visitation and pollen deposition networks in a heathland plant community
Source: Proc Biol Sci. 2015 Sep 7;282(1814):20151130. doi: 10.1098/rspb.2015.1130 (PMC4571695; doi:10.1098/rspb.2015.1130)
Supplement: Floral abundance surveys [file rspb20151130supp1.pdf]

Constructing more informative plant-pollinator networks: visitation and pollen deposition networks in a heathland plant community

G Ballantyne, Katherine C R Baldock, P G Willmer

Electronic Supplementary Material 1

Methods

Floral abundance surveys

Following Forup *et al.* [50], a grid 100 m x 100 m was established approximately 200 m from the heath edge with roughly North-South/East-West axes. Eleven 100 m transects within this were marked out at 10 m intervals. Once a week we randomly selected two 100 m transects and carried out quantitative floral surveys: all individual flowers within a 1 m^2 quadrat were counted at 15 m intervals along each transect, ignoring damaged or faded flowers and buds. In each quadrat 10 flowers of each species present were randomly selected and checked for the presence/absence of pollen (by eye) and of nectar (using 1 µl microcapillary tubes).

Results

Floral phenology and rewards

Patterns of floral abundance and rewards available for each plant species across the three sampling months are shown in Supplementary Information Table 1. *Ulex europaeus* was abundant earlier and flowered through May (although not included in 2013 floral surveys, where fieldwork only began in June). Both *Erica* species were in flower by mid-June, while *Calluna* flowered throughout July and August and *U. minor* began flowering in August. Flowers of *Erica* and *Calluna* are quite long-lived, though older but still attractive flowers only rarely offered pollen or measurable nectar. *Ulex* flowers had no detectable nectar, but many offered pollen when open in May (*U. europaeus*) or in August (*U. minor*). Hence all five species contained many mature but ‘empty' flowers at all times; plants may retain these older long-lived flowers to increase display (though some 'empties' could have been younger flowers depleted by earlier visitation). Many visitors to apparently functional flowers on the heath would thus have received no reward, though they would fare

better if able to restrict their visits to the newly-opened young flowers used for our SVD analysis, where pollen was routinely on offer.

Discussion

There were three distinct seasonal flowering peaks at the study site. *U europaeus* flowers were present in May and early June. *E. tetralix* began to flower at high densities in late June, closely followed by *E. cinerea*. *Calluna vulgaris* was the dominant flowering species in August, with *U. minor* also blooming at a lower density. Consequently our networks present interactions that may be partially separated in seasonal time. However many of the visitor species were recorded throughout the sampling period, and there is considerable overlap in the visitors to each plant species. One exception is the long-tongued *Bombus hortorum*, recorded only on *U. europeaus* in May 2014*.* This may reflect a lack of resources elsewhere at this time of year, with alternatives (e.g. clovers in reasonably nearby fields) becoming available later in the season, so that *B. hortorum* no longer visited the heathland.

Supplementary InformationTable 1. Mean numbers of flowers in 1m^2 quadrats along transects carried out in 2013 (n=12 for each sampling date). Nectar and pollen are given as % of flowers with that reward present; five flowers were sampled in each quadrat, when sufficient flowers were present (n = approx. 50 per sampling period).

**Month & Week**	***Erica tetralix***			***Erica cinerea***			***Calluna vulgaris***			***Ulex minor***		
	Floral density m^{-2}	Nectar	Pollen	Floral density m^{-2}	Nectar	Pollen	Floral density m^{-2}	Nectar	Pollen	Floral density m^{-2}	Nectar	Pollen
June 4	30.9 ± 45.1	-	-	84.2 ± 70.5	-	-	0	-	-	0	-	-
July 1	34.4 ± 31.5	-	-	180.5 ± 134.7	-	-	0	-	-	0	-	-
July 2	44.8 ± 26.4	4.4	18.7	101.1 ± 137.7	2.6	19.1	0	-	-	0	-	-
July 3	28.7 ± 34.0	1.8	18.1	179.4 ± 137.7	2.7	25.7	0.2 ± 0.4	-	-	0	-	-
July 4	12.0 ± 22.2	2.6	18.4	236.8 ± 193.8	0.5	15.9	0.5 ± 1.2	-	-	0	-	-
Aug 1	25.7 ± 29.9	4.0	18.2	136.7 ± 113.9	3.1	8.9	86.7 ± 118.1	2.0	31.2	0	-	-
Aug 2	0	-	-	75.5 ± 135.1	0	9.4	99.2 ± 92.8	0	23.3	7.3 ± 14.7	-	0
Aug 3	10.4 ± 9.7	2.1	22.0	93.3 ± 99.5	0.2	10.3	260.2 ± 280.3	3.6	23.8	1.3 ± 3.8	-	6.0
Aug 4	8.9 ± 14.7	0	15.6	35.2 ± 58.6	0	2.9	136.5 ± 90.0	0	9.0	4.1 ± 9.2	-	26.0
