# Supplementary material for: Constructing more informative plant–pollinator networks: visitation and pollen deposition networks in a heathland plant community
Source: Proc Biol Sci. 2015 Sep 7;282(1814):20151130. doi: 10.1098/rspb.2015.1130 (PMC4571695; doi:10.1098/rspb.2015.1130)
Supplement: Further analysis of pollen deposition by visitors [file rspb20151130supp2.pdf]

Constructing more informative plant-pollinator networks: visitation and pollen deposition networks in a heathland plant community

G Ballantyne, Katherine C R Baldock, P G Willmer

Electronic Supplementary Material 2

Further analysis of pollen deposition by visitors

In addition to the Kruskal-Wallis tests presented in the manuscript, negative binomial GLMs were also carried out on the pollen deposition data.

This GLM and associated post-hoc Tukey tests show that significantly less pollen was deposited on *Erica tetralix* stigmas than all four other plant species. That variation in pollen deposited on the stigmas of different species occurs is not especially surprising given variation in stigma size and morphology, pollen grain size, pollen placement on visitor bodies etc. The model also shows that *Bombus jonellus* deposited significantly more pollen than other visitors, with Tukey tests showing that *B. jonellus* deposited significantly more pollen than *Apis*, *Bombus terrestris/lucorum*, *Lasius*, *Episyrphus*, *Eupeodes*, large syrphids and small syrphids. The tests also show *B. terrestris/lucorum* deposited significantly more pollen than *Apis*. Including an interaction between visitor and plant species in the model was not possible due to complications arising from low sample sizes for many of the combinations.

However, the model is limited in terms of the information we are able to gain from it to answer the question: Does visitor species A deposit significantly more pollen than species B on plant species C? To obtain this information requires the breaking down of the NB into individual negative binomial GLMs with associated Tukey tests for each plant species. Significant differences were found for *E. tetralix* and *U. europeaus*.

On E. tetralix flowers Apis mellifera deposited significantly less pollen than *B. terrestris/lucorum*, *B. jonellus* and *Bombus pascuorum*, while *B. jonellus* deposited significantly more pollen than ants. On *Ulex europeaus* flowers *A. mellifera* deposited significantly less pollen that *B. terrestris/lucorum*, *B. pascuorum*, *B. lapidarius* and other solitary bees.

These results agree with our finding that pollen deposition is more variable on *E. tetralix*, and also highlight the more marginal differences in pollen deposition onto *U. europeaus*. However, the benefits of complicating the analyses with a GLM approach are outweighed by the benefits of the simple, concise Kruskal-Wallis test in the primary manuscript.

Significant differences in Tukey post hoc tests following negative binomial GLMs for pollen depositon onto stigmas

	Tukey post hoc test combination	z-value	p-value
**Plant species**	*E. tetralix – E. cinerea*	8.434	<0.001
	E. tetralix – C. vulgaris	9.781	<0.001
	E. tetralix – U. minor	6.763	<0.001
	E. tetralix – U. europeaus	7.918	<0.001
**Visitor groups for all plant species**	*B. jonellus – A. mellifera*	4.671	<0.001
	B. jonellus – B. terrestris/lucorum	3.680	0.02
	B. jonellus – ants	3.729	0.012
	B. jonellus – large syrphids	3.579	0.02
	B. jonellus – Episyrphus	3.414	0.035
	B. jonellus – Eupeodes	4.404	<0.01
	B. jonellus – small syrphids	4.321	0.01
	B. terrestris/lucorum – A. mellifera	3.760	<0.01
***E. tetralix* visitors**	*B. jonellus – A. mellifera*	5.179	<0.001
	B. jonellus – ants	3.240	0.02
	B. terrestris/lucorum – A. mellifera	5.840	<0.001
	B. pascuorum – A. mellifera	4.486	<0.001
***U. europeaus* visitors**	*B. terrestris/lucorum – A. mellifera*	3.446	0.008
	B. pascuorum – A. mellifera	2.927	0.046
	B. lapidarius – A. mellifera	2.937	0.044
	other solitary bees – *A. mellifera*	3.468	0.008
