# Supplementary material for: Constructing more informative plant–pollinator networks: visitation and pollen deposition networks in a heathland plant community
Source: Proc Biol Sci. 2015 Sep 7;282(1814):20151130. doi: 10.1098/rspb.2015.1130 (PMC4571695; doi:10.1098/rspb.2015.1130)
Supplement: Species level specialisation and species strength for plant species and visitor groups [file rspb20151130supp3.pdf]

Constructing more informative plant-pollinator networks: visitation and pollen deposition networks in a heathland plant community

G Ballantyne, Katherine C R Baldock, P G Willmer

Electronic Supplementary Material 3

Supplementary Information Table: Species level specialisation (d’) and species strength for plant species and visitor groups. The more ecologically relevant metrics are in bold.

	**Visitation**		**Pollinator Effectiveness (SVD)**		**Pollinator Importance**	
	**d'**	**species strength**	**d'**	**species strength**	**d'**	**species strength**
**Plants**						
U. europeaus	0.103	**3.283**	0.316	3.359	**0.118**	3.306
E. tetralix	0.172	**2.707**	0.360	3.240	**0.226**	3.473
E. cinerea	0.152	**2.445**	0.310	2.397	**0.152**	1.757
C. vulgaris	0.244	**4.830**	0.405	5.134	**0.370**	5.464
U. minor	0.378	**2.734**	0.299	1.870	**0.372**	2.001
**Visitors**						
Bombus terrestris/lucorum	**0.080**	2.425	0.027	0.771	0.113	**2.465**
Bombus pascuorum	**0.291**	0.412	0.226	0.520	0.231	**0.435**
Bombus lapidarius	**0.239**	0.632	0.006	0.954	0.261	**0.735**
Bombus jonellus	**0.200**	0.045	0.438	0.611	0.334	**0.156**
Bombus hortorum	**0.363**	0.060	0.406	0.095	0.325	**0.036**
Apis mellifera	**0.213**	0.958	0.174	0.395	0.364	**0.743**
Halictidae	**0.280**	0.078	0.317	0.267	0.216	**0.067**
Other solitary bees	**0.192**	0.159	0.341	0.624	0.273	**0.234**
Large hoverflies	**0.100**	0.029	0.166	0.103	0.135	**0.010**
Episyrphus	**0.206**	0.056	0.304	0.219	0.212	**0.037**
Eupeodes	**0.165**	0.019	0.251	0.073	0.135	**0.005**
Small hoverflies	**0.117**	0.030	0.188	0.132	0.126	**0.008**
Muscidae	**0.350**	0.050	0.468	0.161	0.350	**0.051**
Ants (*Lasius*)	**0.324**	0.035	0.406	0.033	0.281	**0.016**
Lepidoptera	**0.265**	0.012	0.325	0.035	0.105	**0.003**
Soldier fly	**0.105**	0.003	0.239	0.007	<0.001	**<0.001**
