# Supplementary material for: Constructing more informative plant–pollinator networks: visitation and pollen deposition networks in a heathland plant community
Source: Proc Biol Sci. 2015 Sep 7;282(1814):20151130. doi: 10.1098/rspb.2015.1130 (PMC4571695; doi:10.1098/rspb.2015.1130)
Supplement: Network metrics from alternative visitor groupings [file rspb20151130supp4.pdf]

Constructing more informative plant-pollinator networks: visitation and pollen deposition networks in a heathland plant community

G Ballantyne, Katherine C R Baldock, P G Willmer

Electronic Supplementary Material 4

Network metrics from alternative visitor groupings

During data collection hoverflies were identified to genus, but in the analysis the majority were grouped according to size for analyses due to small sample sizes for many of the genera (n < 10 across all plant species). Two genera (*Eupeodes* and *Episyrphus*) had large enough sample sizes to be included at genus level in the networks.

To ensure that the network metrics were not overly influenced by the taxonomic groupings used for visitor groups, we re-analysed the networks twice:

- With the medium sized hoverflies *Episyrphus* and *Eupeodes* grouped together as “medium hoverflies”.
- With all the hoverflies grouped together and with the single soldier fly grouped together with “muscids”.

In both cases the network metrics are very similar to the original results and still follow the same patterns as the existing networks. Values for H_2', weighted nestedness and generality are all still slightly lower in the visitation network than in the PI network than the visitation network than the PI network, and interaction evenness barely changes.

Alternative network metrics for Visitation, Pollinator Effectiveness (PE) and Pollinator Importance (PI) networks when a) hoverfly genera *Episyrphus* and *Eupeodes* are grouped together as “medium hoverflies” b) all hoverfly categories are grouped together under “hoverflies” and the soldier fly visitor is grouped together with “muscids”.

		Network Type		
		Visitation	Pollinator Effectiveness (SVD)	Pollinator Importance
**Metric**				
**H_2'**		0.302	0.330	0.364
**Interaction evenness**		0.672	0.787	0.652
**Weighted nestedness**		0.161	0.059	0.241
**Generality**	**Visitors**	3.773	3.240	3.562
	**Plants**	3.706	6.013	3.385

		Network Type		
		Visitation	Pollinator Effectiveness (SVD)	Pollinator Importance
**Metric**				
**H_2'**		0.299	0.316	0.363
**Interaction evenness**		0.703	0.806	0.686
**Weighted nestedness**		0.126	0.046	0.191
**Generality**	**Visitors**	3.784	3.337	3.570
	**Plants**	3.615	5.445	3.364
